# Progressive Multifocal Leukoencephalopathy in Patients with HIV—Case Series from Northeastern Romania

**DOI:** 10.3390/jcm15031232

**Published:** 2026-02-04

**Authors:** Isabela Ioana Loghin, Marius Gabriel Dabija, Narcis Valentin Tănase, Șerban Alin Rusu, Ion Cecan, Victor Daniel Dorobăț, Carmen Mihaela Dorobăţ, Lucian Eva

**Affiliations:** 1 Department of Infectious Diseases, “Grigore T. Popa” University of Medicine and Pharmacy, 700115 Iasi, Romania; 2 Department of Infectious Diseases, “St. Parascheva” Clinical Hospital of Infectious Diseases, 700116 Iasi, Romaniaion11cecan@gmail.com (I.C.);; 3 Department of Neurosurgery, Clinical Hospital of Emergency “Prof. Dr. Nicolae Oblu”, 700309 Iasi, Romania; 4 Department of Anesthesiology and Intensive Care, “Carol Davila” University of Medicine and Pharmacy, 050474 Bucharest, Romania; 5 Central University and Emergency Military Hospital “Dr. Carol Davila”, 050474 Bucharest, Romania; 6 Department of Intensive Care, National Institute for Infectious Diseases “Prof. Dr. Matei Balș”, 021105 Bucharest, Romania; 7 Department of Neurology, “Dunărea de Jos” University, 800008 Galaţi, Romania

**Keywords:** progressive multifocal leukoencephalopathy, AIDS, human polyomavirus JC, CSF PCR, antiretroviral, MRI

## Abstract

**Background**: Human polyomavirus JC (JCV) causes progressive multifocal leukoencephalopathy (PML), a deadly brain demyelinating illness stemming from oligodendrocyte lytic infection in immunocompromised patients, especially those with untreated HIV infection. **Methods**: We conducted a case series report on patients with HIV/AIDS who presented progressive multifocal leukoencephalopathy and were hospitalized at the “St. Parascheva” Clinical Hospital of Infectious Diseases in Iasi, northeastern Romania, to emphasize the comorbidities of HIV/AIDS cases. Hospital medical data from 10 January 2025 to 30 September 2025 served as the basis for this investigation. **Results**: We examined three cases that presented neurological symptoms (ataxia, aphasia, language comprehension, and expression disorders). The cases were evaluated imagistically via nuclear magnetic resonance, and we conducted a polymerase chain reaction test on the spinal fluid to confirm the presence of JCV. It was necessary to take a multidisciplinary approach with a neurologist or pneumologist. All cases were evaluated immunologically, revealing low Ly T CD4 levels and increased HIV viremia levels. Progressive multifocal leukoencephalopathy is an AIDS-defining disease, manifesting in immunocompromised patients, including late presenter cases, and patients who are non-adherent to their antiretroviral treatment. Therefore, it is important to test every patient who has mild to severe neurological symptoms for HIV. Furthermore, some cases require a multidisciplinary approach to ensure a better quality of life. **Conclusions**: Treating a patient with HIV requires a multidisciplinary strategy that includes a neurology specialist and access to antiretroviral treatment. To boost ART uptake, we must identify and remove barriers that impact patients and the healthcare system.

## 1. Introduction

Since its discovery in 1983, HIV had claimed the lives of over 40.4 million people worldwide as of 2022. This figure is startling, and HIV might develop into a global health emergency without proper vigilance and monitoring. The HIV epidemic has been contained in part by the development and broad accessibility of highly effective antiretroviral treatments (ARTs). Similarly, improvements in opportunistic infections and HIV therapy have made the disease a controllable chronic condition, allowing patients with HIV to lead long, healthy lives. Due to the underlying immunodeficiency, preventing chronic illnesses is a pressing matter for this population [1,2].

The global effort against HIV transmission was announced by UNAIDS, the Joint United Nations Programme on HIV/AIDS. UNAIDS proposed the 90-90-90 aim in 2014, which calls for diagnosing 90% of PLWH globally, providing ART to 90% of PLWH, and achieving viral suppression in 90% of PLWH by 2020 [2]. To achieve prolonged viral suppression, which would stop the progression of the disease, improve morbidity and survival, and lower HIV transmission, the approach comprised detecting HIV infection early, before PLWH could become immunocompromised, and placing them on ART [2]. According to UNAIDS estimates, 90% of PLWH globally had achieved viral suppression by 2020, 84% had received a diagnosis, and 87% had been provided ART. UNAIDS increased the goal to be met by 2025 to 95-95-95, with at least 86% of all PLWH attaining viral suppression, in December 2020 [3,4].

In immunocompromised individuals, JC virus (JCV) reactivation leads to progressive multifocal leukoencephalopathy (PML), a demyelinating disease. Clinical presentation, imaging results, and polymerase chain reaction (PCR) testing for JCV in the cerebrospinal fluid (CSF) are the main factors used for PML diagnosis. Currently, there is no proven treatment for PML. Antiretroviral therapy (ART) is one treatment option that addresses the underlying immunosuppression that hinders the host’s immune response to the virus [5,6].

The goal of ART, the primary treatment for HIV-1-related PML, is to reverse the immunological abnormality that prevents the host from responding normally to JCV. Therefore, this is an “indirect” method of treating PML, but it is the only one that has been effective. Although some antiviral and/or immunomodulatory medications have been suggested or employed as more targeted PML therapies, further investigation has shown that none have been successful [7,8].

Adherence to ART, as well as a comprehensive clinical evaluation and follow-up, may help to improve clinical outcomes and awareness of morbidities [9,10].

This study aims to highlight the critical importance of early HIV and progressive multifocal leukoencephalopathy (PML) diagnoses in young patients, emphasizing the need for prompt and comprehensive neurological evaluation to avoid overlooking associated central nervous system pathology, which, if left undiagnosed and untreated, may rapidly progress and lead to severe disability or death.

## 2. Materials and Methods

### 2.1. Study Design and Database Information

We carried out a case series presentation of three cases of patients with HIV who presented progressive multifocal leukoencephalopathy and were hospitalized at the “St. Parascheva” Clinical Hospital of Infectious Diseases in Iasi, northeastern Romania, to emphasize the comorbidities of HIV/AIDS cases. Hospital medical data served as the basis for our investigation. The research period spanned from 10 January 2025 to 30 September 2025.

Participants were included if they were above the age of 18 and admitted to our Regional HIV/AIDS Center in northeastern Romania after receiving an HIV diagnosis via Western blot (WB) and enzyme-linked immunosorbent assay (ELISA) testing.

During the study period, all adult patients with HIV admitted to our Regional HIV/AIDS Center who presented with new-onset or progressive neurological symptoms were evaluated, and three patients fulfilled the diagnostic criteria for progressive multifocal leukoencephalopathy and were included in this case series. Cerebrospinal fluid analysis, including polymerase chain reaction testing for JC virus DNA, was performed in all patients with suspected PML. In all cases, neurological symptoms preceded hospital admission, followed by brain MRI evaluation, cerebrospinal fluid analysis, and subsequent initiation of antiretroviral therapy.

The purpose of this article is to assess and compile the current developments in managing patients with HIV/AIDS. The debate highlights evidence-based approaches to treating patients by examining contemporary therapy modalities, which tend to improve clinical results and patient quality of life.

The Centers for Disease Control and Prevention (CDC) in Atlanta recommends the use of age-specific CD4+ T-lymphocyte count or CD4+ T-lymphocyte percentage of total CD4 T-lymphocyte cell level to define the HIV infection stage. There are three phases of HIV infection and AIDS: stage 1, which occurs when CD4+ T-lymphocyte counts are more than 500 cells/L; stage 2, which occurs when they are between 200 and 499 cells/L; and stage 3, which occurs when they are less than 200 cells/L. Stages 1 and 2 indicate HIV infection, while stage 3 represents AIDS [1].

### 2.2. Ethical Approval

The “St. Parascheva” Clinical Hospital of Infectious Diseases in Iasi, Romania, granted its approval for the study on 9 January 2025 (Ethical approval no. 1/9 January 2025). Upon admittance, each patient signed a waiver of informed consent.

### 2.3. Study Setting

The primary referral hospital for the Moldova region of Romania is the “St. Parascheva” Clinical Hospital of Infectious Diseases in Iasi, which has 300 beds. There are six pavilions in it. The HIV/AIDS Regional Center and an Infectious Diseases department are located in Pavilion V. With a 12-bed capacity, the Regional Center regularly assesses patients in compliance with EACS and CDC criteria.

The hospital’s central laboratory conducted all blood tests, and the molecular biology lab evaluated the patients’ CD4+ T cell counts and HIV plasma viral loads. RT-PCR HIV-1 was utilized in combination with Cepheid’s GeneXpert^®^ to determine viral load levels and evaluate HIV viremia. If the viral load was less than 40 copies/mL, it was deemed undetectable; if it was greater than 40 copies/mL, it was deemed detectable.

The Regional HIV/AIDS Center in Iasi currently has 1810 patients listed in its active records. To ensure they are following the antiretroviral therapy, patients have a checkup every six months. Each patient has a medical file that contains details about their ART regimens, linked disorders, CD4 T cell count, HIV viral load, and blood test results.

## 3. Results

### 3.1. Case 1

We present the case of a 56-year-old woman with cardiovascular risk factors (arterial hypertension), with neurological APP (right carotid ischemic stroke, left hemiplegia). She presented to the medical emergency service for worsening general condition, worsening motor deficit, and language comprehension and expression disorders. Her symptoms worsened for approximately 3 days before seeking emergency services.

The general physical examination revealed normal cardiovascular parameters, with a blood pressure of 125/78 mmHg, a heart rate of 108 bpm, and rhythmic heart sounds, as well as cardiopulmonary compensation and bilateral pulsatile peripheral arteries.

Objective neurological examination indicated that the patient was conscious but uncooperative, with spatiotemporal disorientation, bradypsychic left hemiplegia (motor deficit in the left limbs, hypotonia of the left hemibody, reduced osteotendinous reflexes on the left), language comprehension and expression disorders of the global aphasia type (echolalia, decreased verbal fluency, does not execute simple or complex orders), central left facial paresis, and cortical blindness.

The patient was admitted to the “N. Oblu” Neurology Hospital for further investigations. A chest CT scan was performed, which revealed right lobe pneumonia. The infectious diseases doctor was contacted, who recommended third-generation cephalosporins and probiotics.

**Figure 1:**
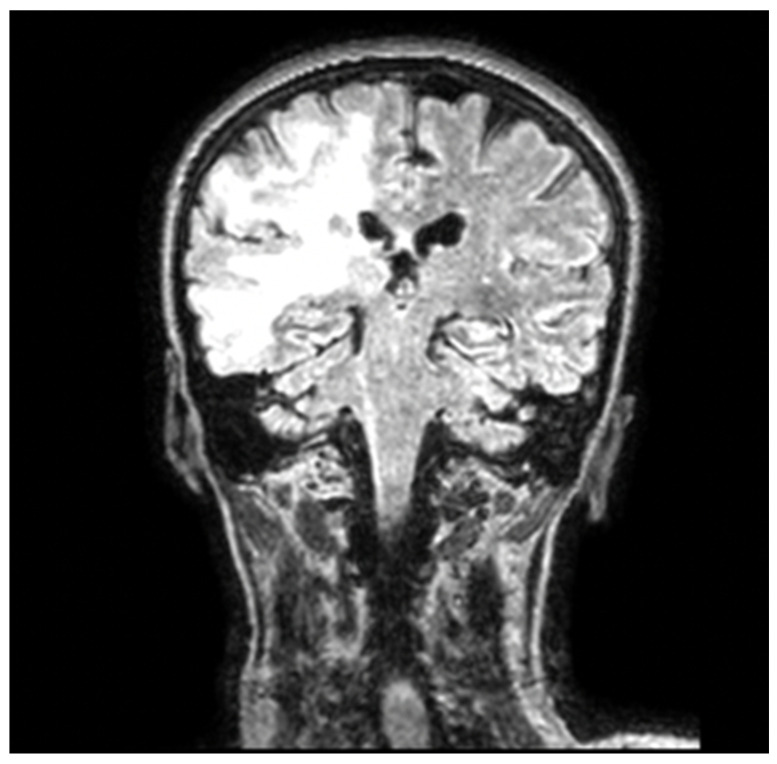
Coronal FLAIR brain MRI showing asymmetric hyperintense lesions in the left parieto-occipital subcortical and periventricular white matter, without mass effect or enhancement, which are findings suggestive of progressive multifocal leukoencephalopathy in an immunocompromised patient.

A craniocerebral MRI with contrast substance was performed, which revealed a wide infiltrative area in the frontotemporal lobes and right insula with extension into the contralateral cerebral hemisphere through the splenium of the corpus callosum (Figure 1). A neurosurgical consultation was performed, with a transfer notice to the neurosurgery department.

A lumbar puncture was performed to collect cerebrospinal fluid, and additional serological analyses yielded a subsequent positive result for polyomavirus JC DNA. HIV serology was also collected (two ELISA positive tests and a further positive Western blot).

Although the neuronavigated brain biopsy showed histopathological features consistent with HIV encephalitis, this finding did not exclude the diagnosis of progressive multifocal leukoencephalopathy. The PML diagnosis in this patient was established based on the typical clinical presentation, characteristic MRI findings of multifocal non-enhancing white matter lesions, and the detection of JC virus DNA in the cerebrospinal fluid by PCR, in accordance with established diagnostic criteria. Brain biopsy in PML has limited sensitivity due to the focal and patchy distribution of demyelinating lesions, and HIV encephalitis and PML may coexist in advanced HIV infection.

It was decided to transfer the patient to the “St. Parascheva” Infectious Diseases Hospital, Iasi, HIV/AIDS Regional Center. Clinically, the patient was aphasic, conscious, uncooperative, and disoriented temporally and spatially, with left hemiplegia, central facial paralysis, pale skin, and mucous membranes. Pulmonary with wheezing rales over the entire bilateral pulmonary area, rhythmic heart sounds, and normal cardiovascular parameters (blood pressure 143/70 mmHg: heart rate 58 bpm, and oxygen saturation 98% in breathing air).

An immunological examination was performed, revealing 64 Ly T-CD4 cells/mm^3^, HIV plasma viremia (600,000 copies/mL). The patient was evaluated for other opportunistic infections, cytomegalovirus infection (Ig M antibodies negative), toxoplasmosis (Ig M antibodies), hepatitis (negative HBc antigen and C hepatitis virus antibodies), and *Treponema pallidum* infection (negative antibodies). Sputum tests and another lumbar puncture were performed to evaluate Mycobacterium tuberculosis infections, with negative results.

During hospitalization, antibiotic treatment consisted of third-generation cephalosporins for 13 days and Trimethoprim/Sulfamethoxazole for pneumocystis pneumonia prophylaxis.

Antiretroviral treatment was initiated cautiously to avoid IRIS (immune reconstitution inflammatory syndrome), according to guidelines, with Darunavir/ritonavir and Emtricitabine/Tenofovir disoproxil fumarate, with good tolerance.

The patient was discharged to the neurologic clinic, with a general stationary afebrile condition, aphasic, uncooperative, and left hemiplegia, central facial paresis, for further investigations and specialized therapeutic conduct.

### 3.2. Case 2

The second case is a 36-year-old patient who was admitted to our Regional HIV AIDS Center in 2007 and diagnosed with HIV infection, stage 3; the patient was non-adherent and non-compliant with ARV therapy with Bictegravir/emtricitabine/tenofovir alafenamide, and was transferred to our center for clinical–biological, viro-immunological investigation and specialized therapeutic treatment.

Before admission to our clinic, the patient presented marked physical asthenia, speech disorders, weakness in the legs and arms that progressively worsened, headache, weight loss, and watery diarrheal stools for approximately 6 days (in remission).

From the patient’s personal pathological history, we noted pulmonary tuberculosis with meningeal affliction, for which the patient followed antituberculous treatment under the supervision of the pneumology specialist.

Clinically, upon admission, he was afebrile, conscious, and cooperative, presenting with the following symptoms: discrete white deposits on the palatine vault, hemodynamic and respiratory stable, blood pressure of 105/75 mmHg, heart rate of 106 bpm, oxygen saturation of 96% in breathing air, pulmonary physiological vesicular murmur, depressed abdomen, sensitive to deep palpation in the epigastrium, intestinal transit, and physiological urination.

A viro-immunological examination was performed, revealing 80 Ly T-CD4 cells/mm^3^ and HIV plasma viremia (1,350,000 copies/mL). The patient was evaluated for other opportunistic infections, cytomegalovirus infection (Ig M antibodies negative), Toxoplasmosis (Ig M antibodies), hepatitis (negative HBc antigen and C hepatitis virus antibodies), and *Treponema pallidum* infection (negative antibodies).

**Figure 2:**
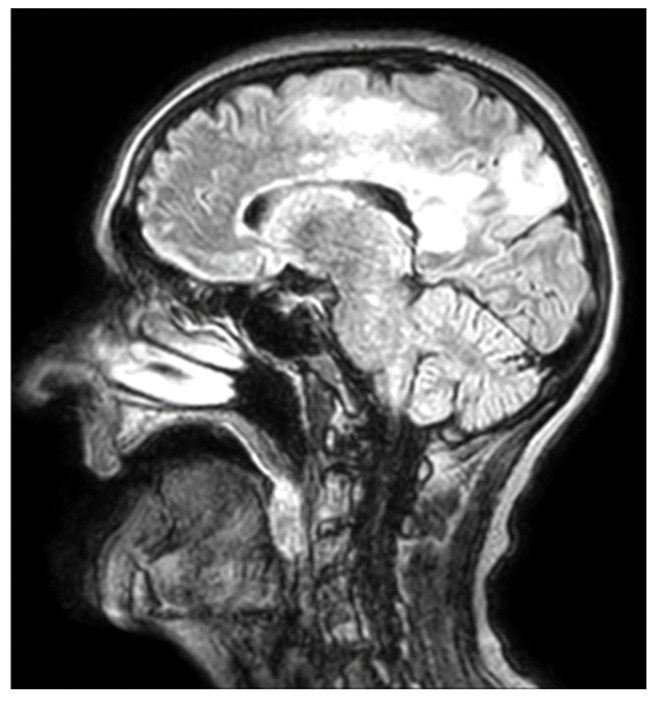
Sagittal FLAIR brain MRI showing a hyperintense lesion involving the left parieto-occipital white matter and extending toward the splenium of the corpus callosum, without mass effect or enhancement, which are findings consistent with progressive multifocal leukoencephalopathy.

Because the patient presented with intense headaches and weakness in the legs and arms that progressively worsened, a neurological exam and craniocerebral MRI were performed. The neurological exam determined that the patient presented light hypotonia of the right hemibody, reduced osteotendinous reflexes on the right, decreased verbal fluency, and mild cortical blindness. The MRI revealed a wide infiltrative area in the temporoparietal lobes (Figure 2).

Lumbar puncture was performed with negative Mycobacterium tuberculosis DNA, but the polymerase chain reaction examination of the spinal fluid revealed positive results for John Cunningham polyomavirus DNA.

During hospitalization, the patient followed the antituberculosis treatment in accordance with the pulmonary recommendations. Antiretroviral treatment was cautiously initiated to prevent immune reconstitution inflammatory syndrome using protease inhibitors and two nucleoside reverse transcriptase inhibitors.

The patient was discharged to the neurological clinic for further investigations and specialized therapeutic care.

### 3.3. Case 3

The third case is a 47-year-old female patient with a history of HIV infection and multiple comorbidities, who was admitted to the Regional HIV/AIDS Center with severe neurological manifestations. The patient had been experiencing progressive motor deficits, gait instability, and speech difficulties over the previous weeks. On admission, she was conscious but disoriented, with significant psychomotor slowing, right hemiparesis with muscle weakness, and motor aphasia-type language impairment. General physical examination showed stable cardiovascular and respiratory parameters.

Neurological assessment confirmed motor deficits predominantly on the right side, diminished osteotendinous reflexes, and language impairment characterized by poor verbal fluency and difficulties in executing simple commands. Immunovirological evaluation revealed a severely immunocompromised status, with a CD4+ T-cell count of 72 cells/mm^3^ and HIV plasma viral load of 850,000 copies/mL.

Because of the specific neurologic symptoms, a cerebral MRI was performed, which revealed brain lesions consistent with progressive multifocal leukoencephalopathy.

A lumbar puncture was performed, and the cerebrospinal fluid polymerase chain reaction (PCR) was positive for John Cunningham polyomavirus DNA.

During hospitalization, empirical antibiotic treatment was initiated for pneumonia, along with prophylaxis for opportunistic infections. Antiretroviral therapy was introduced with a boosted protease inhibitor-based regimen (darunavir/ritonavir combined with emtricitabine/tenofovir disoproxil fumarate), which was well tolerated. A multidisciplinary evaluation by neurology and infectious disease specialists confirmed the diagnosis of progressive multifocal leukoencephalopathy in the context of advanced HIV infection.

The patient’s clinical status showed mild improvement under supportive therapy and ART initiation. At discharge, she was afebrile and hemodynamically stable, but with residual neurological sequelae (motor aphasia, right hemiparesis). She was transferred to the neurology department for further management of acquired neurological sequelae.

## 4. Discussion

PML in the setting of HIV infection significantly occurs in patients with advanced immune suppression. Aye et al. [11] noted that PML is almost exclusively seen in profoundly immunodeficient individuals, with AIDS accounting for over 80% of cases. Similarly, Summers et al. [12] reported that PML typically develops in HIV-positive patients with CD4+ T-cell counts <200 cells/mm^3^, even if they are on antiretroviral therapy (ART). All three patients in our series fit this profile, presenting with CD4 counts well below 200 (Cases 1, 2, and 3 had 64, 80, and 72 cells/µL, respectively), confirming that severe CD4 lymphopenia was a common predisposing factor.

High HIV viremia levels often accompany PML. Hirsch et al. [13] found a median CD4 count of ~90 cells/µL and discovered that over half of PML patients had plasma HIV RNA > 100,000 copies/mL at diagnosis. The main clinical, immunological, and virological features of the three cases are summarized in Table 1, which provides a comparative overview of their baseline characteristics, diagnostic workup, treatment, and outcomes. In our cases, each patient presented an uncontrolled viral load (Cases 1, 2, and 3 had 600,000, 1,350,000, and 850,000 copies/mL, respectively). Notably, Arora et al. also observed that, in ~58% of their cases, PML was the initial AIDS-defining illness (i.e., the first clue to undiagnosed HIV) [14]. In our series, the first case was a patient newly diagnosed with HIV at presentation. The second case was a known patient with poor adherence to ART, who presented an AIDS-defining disease (pulmonary tuberculosis with meningeal affliction), and the third had long-standing HIV infection with late presentation, underscoring how advanced HIV can remain latent until an opportunistic infection triggers it.

These parallels reaffirm that profound immunosuppression—low CD4 counts coupled with active viral replication—sets the stage for JCV reactivation and PML, as evident in our three patients.

Accurate PML diagnosis relies on integrating clinical, radiological, and virological evidence in line with international standards. Falco et al. [15] established consensus criteria indicating that a compatible clinical syndrome, together with characteristic MRI lesions and a positive CSF JC virus PCR result, is sufficient for a definitive PML diagnosis without the need for brain biopsy. We adhered to this approach: all three patients presented with subacute focal neurological deficits and underwent brain MRI, which, in each case, demonstrated the classic multifocal, non-enhancing white matter lesions consistent with demyelination. CSF analysis revealed JCV DNA by PCR, confirming the diagnosis.

This pattern aligns with the observations of Falco et al. [15] and Engsig et al. [16], who emphasized that MRI and JCV-PCR are the cornerstones of diagnosis. Importantly, in all three cases, other opportunistic infections such as CMV, toxoplasmosis, hepatitis, syphilis, and tuberculosis were systematically excluded through serology, cultures, and PCR testing, reinforcing the PML diagnosis. In Case 1, a neuronavigated brain biopsy also demonstrated HIV encephalitis, emphasizing that PML may coexist with other HIV-related neuropathological changes.

There is no specific antiviral cure for PML, so management hinges on optimizing ART to restore immune function. Antinori et al. emphasized that, to date, no targeted anti-JC virus therapy has proven effective and that HAART remains the only proven effective treatment for AIDS-related PML [17]. All three of our patients received prompt ART initiation or regimen escalation as the cornerstone of therapy. All cases received protease inhibitor-based regimens (darunavir/ritonavir combined with emtricitabine/tenofovir disoproxil fumarate), showing stabilization and survival. The literature supported the rationale for including potent agents. For example, in a Spanish multicenter cohort, French et al. [18] observed that patients with PML on protease inhibitor-inclusive regimens had significantly lower 1-year mortality than those on regimens without protease inhibitors.

In Case 2, the switch from BIC/FTC/TAF to a protease inhibitor-based regimen (DRV/r + FTC/TDF) was not driven by documented antiretroviral drug resistance, as resistance testing was not available at the time of regimen change. Rather, the decision was based on clinical considerations. In patients with poor adherence to an INSTI-based regimen, there is an increased risk of rapid resistance development, potentially compromising the entire integrase inhibitor class. Darunavir boosted with ritonavir has a high genetic barrier to resistance and maintains virologic efficacy despite intermittent adherence. In the context of advanced HIV infection, high viral load, low CD4 count, and PML, a protease inhibitor-based regimen was therefore preferred as a more robust and forgiving therapeutic option while adherence issues were being addressed.

Our findings confirm that timely ART remains the cornerstone of PML therapy, and the possibility of immune reconstitution inflammatory syndrome should always be considered, as highlighted by Tan and McArthur [17]. None of our cases developed severe PML-IRIS.

The prognosis of HIV-associated PML has improved in the ART era compared to the universally grim outcomes of the pre-HAART era. However, mortality remains high, and survivors are often left with significant neurological sequelae. Historically, without therapy, median survival was only about 3–4 months [13]. With the advancement of HAART, one-year survival rates have increased substantially; for instance, Shah et al. [19] note that median survival after PML diagnosis extended from ~6 months pre-1996 to over 2 years in the contemporary era.

In a European cohort, Berenguer et al. reported that PML patients with baseline CD4 counts < 100/µL had a 2.8-fold higher risk of death [10], underscoring the importance of immune status at presentation. Consistent with those findings, Cases 1 and 2 survived but with significant neurological deficits, while Case 3 experienced partial clinical improvement and survived with neurological sequelae. Two of our three patients (66%) are alive at one-year follow-up, which is in line with recently reported survival rates of approximately 50–70% on effective ART [12,13]. This modest improvement in mortality relative to pre-HAART times reflects what Yoganathan et al. described—namely, better prognoses are seen in those who demonstrate immune recovery (i.e., rising CD4 counts and clearance of JCV from CSF) on therapy [20]. Even so, PML remains a life-threatening illness.

In a Portuguese series with suboptimal ART, mortality was as high as 81% [12], whereas in more recent cohorts with widespread ART use, death rates around 40–50% are reported [13]. Our case series outcomes fall within this improved range yet highlight that vigilance is needed.

Importantly, surviving PML often entails chronic neurologic deficits. Khanna et al. [21] observed that patients who survive PML frequently have permanent neurological sequelae corresponding to the areas of brain demyelination. A systematic review by Aye et al. [11] similarly concluded that a large proportion of HIV-PML survivors suffer long-term impairments in motor, visual, or cognitive function. Consistently, in a Brazilian case series, 42.8% of PML survivors were left with significant sequelae, such as limb weakness, ataxia, speech difficulties, or visual loss [12]. This trend was also seen in our patients: Case 1 remained with global aphasia and hemiplegia, Case 2 with residual motor and cognitive deficits, and Case 3 with motor aphasia and right hemiparesis. Nonetheless, these patients have stabilized neurologically and are living with these deficits, which is a better outcome than the near-certain fatality seen in the pre-ART era.

Alstadhaug et al. [22] reported that aggressive ART not only prolongs survival but can also lead to JCV clearance from CSF and partial neurological recovery, an effect we observed as well: both surviving patients in our series showed some improvement (or at least no progression) in their neurologic status once virological suppression was achieved. Overall, our three-patient series aligns with published case series and cohort studies demonstrating that early ART-driven immune restoration can convert PML from an acutely fatal disease to a chronic, manageable condition—albeit one often accompanied by considerable neurologic disability [22,23].

For successful results, HAART and early diagnosis are crucial. Clinicians should have a low threshold of suspicion for diagnosing PML-IRIS in any patient with HIV with unexplained neurological symptoms, regardless of the CD4 cell level [24,25]. However, those who survive PML-IRIS with neurological sequelae might benefit from extensive, multimodal rehabilitative therapy [26,27].

Because neurological manifestations in advanced HIV infection may overlap with other opportunistic conditions, a differential diagnosis should be carefully considered. Table 2 summarizes the main alternative diagnoses and the criteria used to distinguish them from PML in our cases.

The observed outcomes (66% survival, with survivors experiencing moderate sequelae) are comparable to those in other contemporary reports of HIV-related PML [12,13]. These cases underscore the critical importance of prompt PML recognition, MRI and CSF-based diagnosis, exclusion of other opportunistic infections, and immediate initiation of effective ART. By comparing our findings with the broader literature, we conclude that our patients’ clinical courses reflect the current international experience: combination antiretroviral therapy has significantly improved PML survival, yet PML diagnosis still portends high mortality and enduring morbidity, emphasizing the need for continued research into adjunctive treatments to improve outcomes of this devastating opportunistic CNS infection. The main limitation of our report is the small number of cases and lack of long-term follow-up imaging; however, the clinical and virological data provide valuable insights into the management and prognosis of HIV-related PML.

## 5. Conclusions

Given patient-level differences, the clinical results of PML in patients with HIV/AIDS raise medical concerns. The illness manifests as a variety of pathogenic processes. Medical therapies to prolong life and rehabilitation efforts to enhance functional status and quality of life in PML can be made possible by the right diagnosis. Even with the use of HAART, PML is still linked to a high rate of morbidity; however, early ART initiation dramatically lowers mortality.

HAART and early diagnosis are crucial. Clinicians should have a low threshold of suspicion for diagnosing PML-IRIS in any HIV-infected patient with unexplained neurological symptoms, regardless of the CD4 cell level. In rare cases, even with therapeutic care, the illness can advance quickly and cause death in a matter of weeks. Therefore, it is critical to recognize neurological pathology in an immunodeficient context, considering that the standard of treatment for HIV-associated PML is HAART to achieve immunological recovery and optimal virological control of HIV. PML prognosis has improved considerably since the development of HAART. However, those who survive PML-IRIS with neurological sequelae might benefit from extensive, multimodal rehabilitative therapy. The prognosis of our patients was favorable upon discharge; nevertheless, though such a condition is not common, it should serve as a warning for doctors and patients with suspected PLM and AIDS and represent an alarm signal for testing even asymptomatic patients.

## Figures and Tables

**Table 1:** Clinical and virological characteristics of the three PML cases.

Characteristic	Case 1	Case 2	Case 3
Age/Sex	56/F	36/M	47/F
Comorbidities	Hypertension, stroke	Non-adherence, Pulmonary TB with meningitis affliction	Long-standing HIV
CD4 (cells/mm^3^)	64	80	72
HIV RNA (copies/mL)	600,000	1,350,000	850,000
Main neurological signs	Global aphasia, hemiplegia, blindness	Headache, weakness, mild cortical blindness	Motor aphasia, right hemiparesis
Imaging	MRI: frontotemporal lesions	MRI multifocal lesions	MRI: multifocal lesions
CSF PCR (JCV)	Positive	Positive	Positive
Other OIs ruled out	Yes	Yes	Yes
ART initiated	DRV/r + FTC/TDF	BIC/FTC/TAF- nonadherence, then DRV/r + FTC/TDF	DRV/r + FTC/TDF
Outcome	Survived, sequelae	Survived, sequelae	Partial improvement, survived with sequelae

**Table 2:** Differential diagnosis of neurological manifestations in HIV-positive patients.

Condition	Imaging	CSF Findings	Clinical Context
PML (JCV)	Multifocal, non-enhancing	JCV-PCR positive	CD4 < 200, progressive symptoms
Toxoplasmosis	Ring-enhancing lesions	IgG/IgM, PCR positive	Often single/few lesions
TB meningitis	Meningeal enhancement	TB-PCR positive	Headache, fever, systemic TB
CMV encephalitis	Ventriculoencephalitis	CMV-PCR positive	Very low CD4, retinitis
HIV encephalitis	Diffuse atrophy/lesions	Usually, negative	Chronic progressive dementia

## Data Availability

The original contributions presented in this study are included in the article. Further inquiries can be directed to the corresponding authors.
